# A handbook for junior doctors on the medical management of eating disorders on an inpatient unit

**DOI:** 10.1186/2050-2974-3-S1-O5

**Published:** 2015-11-23

**Authors:** Georgina Brunton, Warren Ward

**Affiliations:** 1 Royal Brisbane Women's Hospital, Herston, QLD, Australia

## 

Eating Disorders are serious mental illnesses with potentially life-threatening physical morbidities and high mortality rates. Despite this, the medical management of these patients on an inpatient setting is often left to the care of junior medical staff, who, while often well trained in the medical complications of obesity, have seldom had training in the risks and management of malnutrition secondary to starvation and other related complications seen in Eating Disorders. This can sometimes result in under-treatment, misinformation given to patients, and even premature discharge of the seriously unwell patient.

In this presentation, the authors will describe the content of a handbook developed for junior medical staff in the acute hospital setting, to guide the management of common medical complications in physically compromised patients with Eating Disorders. It provides practical treatment advice based on current guidelines, as well as explaining the rationale for medical monitoring in a simple and easy to use format. Additionally, it also aims to guide medical staff on how to explain to the patient the findings of medical investigations and examinations and how these findings relate to their eating disorder.

